# The Dubousset Functional Test: a reliable and valid test in early stage Parkinson’s disease patients

**DOI:** 10.1007/s10072-024-07359-1

**Published:** 2024-02-01

**Authors:** Ayşe Abit Kocaman, Saniye Aydoğan Arslan, Yusuf Emre Bozkurt, Erdal Coşkun

**Affiliations:** 1 Faculty of Health Sciences, Department of Physiotherapy and Rehabilitation, Kırıkkale University, Kırıkkale, Turkey; 2 Samsun Physical Medicine and Rehabilitation Diseases Hospital, Samsun, Turkey; 3 Department of Physical Medicine and Rehabilitation, Sincan Medical Center, Ankara, Turkey

**Keywords:** Dubousset Functional Test, Outcome measures, Parkinson’s disease, Physical function, Validity, Reliability

## Abstract

**Introduction:**

Dubousset Functional Test (DFT) is an assessment test evaluating the functional capacity and dynamic balance. The study aimed to examine the reliability, validity, and responsiveness of the DFT in early stage Parkinson’s disease (PD) patients.

**Methods:**

This was a cross-sectional study. Thirty-three early stage PD patients were recruited. The DFT was performed along with the Timed Up and Go (TUG) test, dual-task TUG, Functional Reach Test (FRT), 3-m backward walk test (3MBWT), Tinetti Performance-Oriented Mobility Assessment (POMA), and Berg Balance Scale (BBS).

**Results:**

The test–retest reliability of the subcomponents of the DFT was excellent. The ICCs were as follows: 0.952, 0.955, 0.917, and 0.919, respectively. The correlation with subcomponents of DFT and TUG, dual-task TUG, FRT, 3MBWT, BBS, and POMA was found to be statistically significant (*p* < 0.05). The standard measurement errors of the subcomponents of the DFT were 1.45, 1.39, 1.70, and 1.57, respectively. The minimal clinically important difference (MCID) of the subcomponents was 2.05, 1.97, 2.41, and 2.22, respectively.

**Conclusion:**

The DFT is a reliable, valid, and easy-to-administer tool in assessing the balance and physical function of early stage PD patients.

## Introduction

Parkinson’s disease (PD) is an age-related neurodegenerative disease characterized by slowdown of movement (bradykinesia), muscle stiffness (akinesia), resting tremor, and loss of postural reflexes. Motor and non-motor symptoms lead to numerous clinical symptoms associated with movement disorder [1]. Parkinson’s patients usually have forward flexed posture. The knee, the hip, the head, the neck, and the trunk are in flexion, and the shoulders are in protraction. A significant part of patients have more serious spinal deformities such as excessive forward flexion of the head and neck (antecollis), excessive thoracolumbar flexion (camptocormia), excessive lateral flexion of the spine (Pisa syndrome), and scoliosis [2]. Moreover, rigidity in the global trunk muscles decreases the spinal mobility of patients and influences the measures of independence in trunk-dependent activities. Meanwhile, postural correction and balance reactions decrease in people with Parkinson’s disease (PwPD) because of postural changes such as the development of axial rigidity and reduced trunk rotation [3]. On the other hand, deformities and axial rigidity in the spinal region usually cause a decrease in walking ability [2], postural instability, participation in activities of daily living (e.g., getting up from a chair, climbing stairs), quality of life, recurrent falls, fall-related injuries, fear of a secondary fall, and balance disorder that adversely affects functional mobility [4, 5]. It is reported that the rate of falls is higher in PwPD (50%) compared to healthy older adults (15%) [6]. In PwPD, about 75% of falls result from the inability to control body mass due to axial rigidity during activities of daily living, such as standing, leaning forward, and turning. The appropriate control of trunk movement is extremely important for postural stability since the upper part of the body is responsible for two-thirds of the body weight and the center of gravity [7]. Even small, uncoordinated movements of the trunk may increase the fall risk and loss of balance because of the height of the center of gravity of the body from the ground and the heavy mass of the body [8]. Therefore, balance evaluations performed in PwPD determine the fall risk and are quite valuable in preventing complications that may emerge due to loss of balance. On the other hand, in PwPD, impairments in both mobility and cognition are common, and more attention is required in movements that have previously been automatic [9].

Imaging studies on the healthy brain have demonstrated that, as a movement becomes automatic, brain activity in the dorsolateral prefrontal cortex and anterior cingulate cortex decreases, whereas there is an increase in the connection between the putamen and different motor areas. However, such an increase in connection does not occur due to dopamine depletion in the putamen in PwPD, which results in difficulties acquiring automaticity [10]. As a result, they perform a specific task using sources of attention more. A motor-cognitive task involves performing two independent tasks, such as answering arithmetic questions while walking, simultaneously. Performing motor-cognitive tasks requires the involvement of both motor and cognitive systems [11]. This dual-task action is, by definition, carried out by performing two tasks with different objectives simultaneously [12]. Motor-cognitive task performance is reduced in PwPD compared to healthy control individuals matched in terms of age, gender, and education [11].

A lack of dual tasks is defined as a reduction in motor or cognitive performance (or both) when tasks are performed simultaneously [13, 14]. Studies have revealed that dual-task balance is under the control of high-level cognitive processes related to attention and executive functions [15, 16]. Depending on basal ganglia pathology, both executive function and attention disorders are observed even at the early stages of Parkinson’s disease. Hence, PwPD appear to be disproportionately influenced by the dual-task balance compared to their peers of the same age [17]. Thus, achieving dual-task balance can increase dependence on cognitive resources to optimize motor control. Lack of dual tasks causes balance disorders and is a significant sign of PD since it may lead to an increased fall risk [18]. Therefore, early identification of dual-task balance disorders is important to determine the fall risk in PwPD.

In the literature, there are many scales evaluating balance and mobility in PwPD and functional performance tests such as forward reach tests [19], Timed Up and Go (TUG) test [20], Tinetti Performance-Oriented Mobility Assessment (POMA) [21], Berg Balance Scale (BBS) [22], and Balance Evaluation Systems Test (BESTest) [23]. Most balance evaluation scales used in PwPD evaluate static and dynamic sitting balance while standing or only dynamic balance. Since balance is a dynamic process that gradually changes in PwPD, there is a need for measurement methods that can be quantified to record changes and determine the appropriate treatment for these variables.

The Dubousset Functional Test (DFT) was developed by Dr. Jean Dubousset as a practical four-component evaluation test to assess the physical function and balance capacities of individuals with spinal deformities. The DFT consists of four components: getting up from a chair without arms and walking 5 m forward and backward, ascending-descending the steps, transition from a standing position to a sitting position, and the test in which gait is evaluated with the individual’s dual-task test (while counting down from 50) [24]. The difference of this test from other tests is that it evaluates the functionality of spinopelvic muscle groups that are directly correlated with maintaining global trunk smoothness and provides objective results about the individual’s functional performance and balance level. Moreover, unlike other functional performance tests, it measures the individuals’ sufficient coordination, balance, attention, and thinking skills during the function with the dual-task test component to evaluate the neurophysiological process, which requires the person to perform two tasks simultaneously.

When the literature was reviewed, no validity and reliability study of the Dubousset Functional Test in PwPD, which was developed to evaluate functional performance and balance in individuals with spinal deformity. The Dubousset Functional Test is an easily applicable, comprehensible scale that takes a short time and yields objective results in individuals with spinal problems. In line with this information, we assume that the DFT will provide an effective evaluation in revealing functional performance and balance problems in the early period of clinical use in PwPD and help to take necessary precautions. Hence, the aim of the present study is to investigate the validity and reliability of the Dubousset Functional Test in early stage Parkinson’s disease (PD) patients, question the effectiveness of its clinical use, and bring it to the use of other researchers.

## Methods

### Participants

This cross-sectional study was approved by the Kırıkkale University Non-Interventional Research Ethics Committee with the decision number 2020.11.16. The trial was registered at ClinicalTrials. gov (NCT04622657). Written informed consent had been obtained from all the participants before the study started. This study was conducted in accordance with the Declaration of Helsinki.

The inclusion criteria were as follows: participants who were clinically diagnosed as idiopathic PD that fulfilled the UK Brain Bank criteria [25] were included in the study. PwPD were included if they (1) were at least 40 years old, (2) in Hoehn and Yahr (H&Y) stage 1–4, and (3) and able to walk at least 10 m independently with or without an assistive device. They were excluded if they had (1) any neurological conditions other than PD; (2) any vestibular, musculoskeletal, orthopedic, and cardiovascular diseases that might influence balance, or (3) a Mini-Mental State Examination score of < 24 [26]; (4) had any comorbidity that would hinder proper assessment; (5) receving exercise training in the last 6 months. All participants were tested while on anti-parkinsonian medication.

### Sample size calculation

According to the recommendation for reliability analyses, 30–50 participants should be included in the study [27]. Post hoc power analysis was performed with the G * Power program (version 3.0.10 Universität Düsseldorf, Düsseldorf, Germany) to determine the power of the study. In post hoc power analysis, when the statistical significance of alpha was 5% and the confidence interval was 95%, the power of the study (1-β) was found to be 99% for 33 people with Parkinson’s disease. The effect size for the intrarater was calculated as 0.731. The primary outcome was determined as DFT up and walking test and TUG.

### Outcome measures

#### The Dubousset Functional Test (DFT)

The Dubousset Functional Test (DFT) consists of four components.

- **Up and walking test:** The individual gets up from the chair without armrests without assistance, walks 5 m, walks backward without turning back, comes back to the chair, and sits again on the chair without assistance. The time elapsed in the meantime is recorded in seconds. In the test, the start marker is placed 30 cm from the chair. A second marker is placed 5 m (500 cm) from the first marker. Participants were instructed to not pass the second marker
- **Steps test:** The starting position begins by standing 50 cm away from the step. Three steps are climbed, turned around on the third step and three steps are descended by going back. The time elapsed in the meantime is recorded in seconds.
- **Down and sitting test:** The individual moves from the standing position to the sitting position from the ground and then returns to the standing position. He/she uses an assistive device. The time elapsed in the meantime is recorded in seconds.
- **Dual-tasking test:** The individual walks 5 m forward and then turns back to the starting position. He/she counts down from 50 by intervals of 2. The time elapsed in the meantime is recorded in seconds [24]. The markers in the up and walking test were also used in the dual task test. Participants begin the test from a standing position behind the first marker. Participants were instructed to not pass the second marker.

#### The TUG

TUG is designed as a tool for assessing dynamic balance, gait speed, and mobility. Completing the test requires participants to stand up from a chair with armrests, walk 3 m, turn around, walk back to the chair, and sit down. The time taken to complete the test is recorded using a stopwatch [20]. The TUG has demonstrated excellent test–retest (ICC ¼ 0.80) reliability in PwPD [28].

#### Dual-task TUG (additional cognitive task)

During the TUG test, we used a counting backwards from 50 by two as an additional cognitive task. When the test had to be repeated, individuals continued to count from the number they stayed at. Counting errors were ignored [29].

#### The Tinetti POMA

The Tinetti POMA, also called the Tinetti Mobility Test, is a reliable and valid clinical test used to measure balance and gait abilities. The total POMA scale (POMA-T) comprises of a balance subscale (POMA-B) and a gait subscale (POMA-G). The maximum possible total score for POMA-T is 28, for POMA-B is 16, and for POMA-G is 12. Interrater and intrarater reliability of POMA was good to excellent (intraclass correlation coefficient of > 0.80) in PwPD [21].

#### The Berg Balance Scale (BBS)

The Berg Balance Scale (BBS) is widely used as an assessment tool for functional balance. The BBS has 14 items and each item is scored from 0 to 4 according to the level of balance impairment. Higher scores of BBS indicate better balance performance. It has excellent interrater (ICC ¼ 0.98) and test–retest reliability (ICC ¼ 0.95) in PwPD [22].

#### The 3-m backward walk test (3MBWT)

A distance of 3 m (m) is measured and marked with black tape. Patients were asked to follow the black band with their heels. With the “start” command, they are asked to walk backward quickly. When the 3-m distance is completed, they were asked to stop. The evaluator walked behind the individuals throughout the test. The measurements were repeated three times, and the averages were recorded [30]. The 3MBWT demonstrated excellent test–retest reliability (ICC = 0.965) in PwPD [31].

#### Functional Reach Test (FRT)

The Functional Reach Test (FRT) is a test of dynamic bilateral stance balance. At the beginning of the test, the dominant arm is flexed 90° and the distance between the feet is 10 cm. The maximum distance that a person can extend his arm horizontally forward without moving his feet is measured in centimeters. The maximum distance they could reach and return to their former position without losing their balance was measured. The test was repeated three times, and these three values were averaged [19]. The FRT has also been validated in PwPD, with Behrman et al. [32] demonstrating that a cutoff reach score of 25.4 cm accurately individuals at high risk for falling (specificity ¼ 92%, positive predictive value ¼ 90%).

#### The Unified Parkinson’s Disease Rating Scale (UPDRS)

The Unified Parkinson’s Disease Rating Scale (UPDRS) is for clinical assessment of disease severity in PwPD. It consists of four parts: I—mentation, behavior, and mood; II—activities of daily living; III—motor symptoms; and IV—complications of therapy [33].

#### The Hoehn and Yahr (H&Y) Scale

The Hoehn and Yahr (H&Y) Scale is a commonly used system for describing how the symptoms of PD progress. It categorizes from 1 to 5, and higher stage indicates more severe disability [34].

### Testing procedures

The demographic data were recorded at the baseline assessment. Our study was conducted as “test–retest” design and the psychometric properties of Dubousset Function Test (DFT) were examined in PwPD. The DFT, TUG, dual-task TUG, Tinetti Performance-Oriented Mobility Assessment (POMA), Berg Balance Scale (BBS), Functional Reach Test (FRT), and 3-m backward walk test (3MBWT) were applied to the patients. The completion times of the DFT were recorded by the same physiotherapist. To establish the reliability of the DFT, the second evaluation (retest) was carried out by the same physiotherapist 7 days following the first evaluation (test). It was preferred to collect data with a same physiotherapist in order to avoid the interrater variability error rate between the evaluations. The 2-min rest periods were allowed between assessments to minimize fatigue effects. Participants did not receive any treatment for 7 days and were evaluated for test–retest at the same time of day.

### Statistical analysis

Data analysis was conducted using the SPSS 23.0 (SPSS Inc., Chicago, IL, USA) program. The Kolmogorov–Smirnov test was used to check the normality of the distribution of variables. Numerical variables were presented as mean and standard deviation (SD) and categorical variables as frequency percentage (%) in descriptive analysis. Statistical significance was accepted as *p* < 0.05.

### Reliability

The Cronbach’s alpha reliability coefficient and test–retest reliability and intraclass correlation coefficient (ICC) were utilized for internal consistency in reliability analyses. The ICC coefficient was considered as weak if it was smaller than 0.40, as below moderate if it was between 0.40 and 0.59, as moderate if it was between 0.60 and 0.74, as good if it was between 0.75 and 0.89, and as very good if it was greater than 0.90 [35].

### Validity

Convergent validity analysis was used to investigate the validity of the DFT in early stage PD patients. For convergent validity, the relationship between the TUG, dual-task TUG, POMA, BBS, FRT, UPDRS-III, and 3MWBT was evaluated with the Spearman correlation test. Dancey and Reidy’s classification was used to decide on the strength of the correlation: 0.00 indicates no correlation, 0.001–0.29 low-level correlation, 0.30–0.70 moderate-level correlation, 0.71–0.99 high-level correlation, and 1.00 indicates the perfect correlation [36]. Standard error of measurement (SEM) and minimal detectable change (MDC): SEM is an estimate of random variation that occurs in data without any real changes. It can be calculated from MDC and SEM with 95% accuracy. The MDC value is defined as the minimum amount of change that must be observed in the data, either as a group or individually. In our study, the SEM and MDC values were computed for the DFT. It was calculated using the following formula: MDC95% = 1.96 * SEM * √2; SEM = SD√ (1 − ICC) [37].

## Results

This study included a total of 33 early stage PD patients (20 men, 13 women; mean age, 68.63 ± 8.72). The sociodemographic and clinical data of the participants are presented in Table 1.

**Table 1:** Participants’ characteristics

Variables	People with Parkinson’s disease (*n* = 33)
Age (years) mean ± SD	68.63 ± 8.72
BMI (kg/m^2^) mean ± SD	27.52 ± 4.00
Gender	
Female, *n* (%)	13 (39.4)
Male, *n* (%)	20 (60.6)
MMSE score, mean ± SD	24.81 ± 2.03
Dominant side, *n* (%)	
Right	32 (97)
Left	1 (3)
Disease duration (years)	5.09 ± 3.39
H&Y stage, *n* (%)	
1.5	7 (11.5)
2	7 (11.5)
2.5	4 (6)
3	15 (24.6)
FRT (cm) *x* ± sd	13.73 ± 6.65
BBS (score) *x* ± sd	35.84 ± 8.67
3MBWT (s), *x* ± sd	11.95 ± 4.74
UPDRS-III (score), *x* ± sd	22.66 ± 6.92
POMA-G (score) *x* ± sd	3.51 ± 1.54
POMA-B (score) *x* ± sd	15.39 ± 4.06
POMA-T (score) *x* ± sd	18.90 ± 5.04
TUG (s) *x* ± sd	16.53 ± 6.12
Dual-task TUG (s) *x* ± sd	19.72 ± 7.41
DFT up and walking test (s) *x* ± sd	20.90 ± 6.76
DFT step test (s) *x* ± sd	16.98 ± 6.37
DFT down and sitting test (s) *x* ± sd	15.19 ± 5.83
DFT dual-tasking test (s) *x* ± sd	21.46 ± 5.12

*x* ± *sd*, mean ± standard deviation

*n*, participant; *%*, percentage

*BMI*, body mass index; *DFT*, Dubousset Functional Test; *FRT*, Functional Reach Test; *BBS*, Berg Balance Scale; *3MBWT*, 3-m backward walk test; *UPDRS-III*, Unified Parkinson’s Disease Rating Scale-motor function; *POMA-G*, Tinetti Performance-Oriented Mobility Assessment-Gait; *POMA-B*, Tinetti Performance-Oriented Mobility Assessment-Balance; *POMA-T*,Tinetti Performance-Oriented Mobility Assessment-Total; *TUG*, Timed Up and Go Test

### Reliability analysis

The test–retest reliability ICC values were found to be 0.952 for the DFT up and walking test, 0.955 for the DFT step test, 0.917 for the DFT down and sitting test, and 0.919 for the DFT dual-tasking test. For intrarater agreement, ICC values on the individual tests were between 0.917 and 0.955 (excellent agreement) (Table 2).

**Table 2:** The relative (ICC coefficient) and absolute (SEM and MDC95) reliability of the DFT

	ICC (2,1)	%95 CI	SEM	MDC_95%_
Up and walking test	0.952	0.903/0.976	1.45	2.05
Step test	0.955	0.908/0.978	1,39	1.97
Down and sitting test	0.917	0.833/0.959	1.70	2.41
Dual-tasking test	0.919	0.837/0.960	1.57	2.22

*DFT*, Dubousset Function Test; *CI*, confidence interval; *ICC*, intraclass correlation coefficient; *SD*, standard deviation; *SEM*, standard error of measurement with a 95% CI; *MDC*_*95%*_, minimum detectable change at 95% of confidence interval

In this study, the SEM and MDC values of the DFT up and walking test were 1.45 and 2.05, the SEM and MDC values of the DFT step test were 1.39 and 1.97, the SEM and MDC values of the DFT down and sitting test were 1.70 and 2.41, and the SEM and MDC values of the DFT dual-tasking test were 1.57 and 2.22, respectively (Table 2).

### Validity analysis

A high level correlation was found between the DFT up and walking test and BBS, 3MBWT, TUG, and dual-task TUG, and a moderate correlation was determined between the POMA-G, POMA-B, POMA-T, UPDRS-III, and FRT. A high level correlation was revealed between the DFT step test and the 3MBWT, TUG, and dual-task TUG and a moderate correlation was identified between the FRT, BBS, and POMA-G, POMA-B, POMA-T, and UPDRS-III. A high level correlation was observed between the DFT down and sitting test and the dual-task TUG and a moderate level correlation was determined between BBS, TUG, 3MBWT, POMA-G, POMA-B, POMA-T, UPDRS-III, and FRT. A high level correlation was found between the DFT dual-tasking test and 3MBWT, TUG, and dual-task TUG, and a moderate correlation was identified between the FRT, BBS, POMA-G, POMA-B, POMA-T, and UPDRS-III (Table 3).

**Table 3:** Correlation between the DFT and other outcome measures in people with Parkinson’s disease

	FRT	BBS	3MBWT	UPDRS-III	POMA-G	POMA-B	POMA-T	TUG	Dual-task TUG
Up and walking test	*r* = − 0.698 *p* = 0.001	*r* = − 0.753 *p* = 0.001	*r* = 0.850 *p* = 0.001	*r* = 0.533 *p* = 0.001	*r* = − 0.492 *p* = 0.004	*r* = − 0.589 *p* = 0.001	*r* = − 0.639 *p* = 0.001	*r* = 0.732 *p* = 0.001	*r* = 0.765 *p* = 0.001
Step test	*r* = − 0.686 *p* = 0.001	*r* = − 0.646 *p* = 0.001	*r* = 0.704 *p* = 0.001	*r* = 0.444 *p* = 0.010	*r* = − 0.634 *p* = 0.001	*r* = − 0.518 *p* = 0.002	*r* = − 0.623 *p* = 0.001	*r* = 0.810 *p* = 0.001	*r* = 0.746 *p* = 0.001
Down and sitting test	*r* = − 0.586 *p* = 0.001	*r* = − 0.422 *p* = 0.015	*r* = 0.497 *p* = 0.003	*r* = 0.396 *p* = 0.023	*r* = − 0.340 *p* = 0.053	*r* = − 0.529 *p* = 0.002	*r* = − 0.518 *p* = 0.002	*r* = 0.687 *p* = 0.001	*r* = 0.703 *p* = 0.001
Dual-tasking test	*r* = − 0.698 *p* = 0.001	*r* = − 0.605 *p* = 0.001	*r* = 0.773 *p* = 0.001	*r* = 0.333 *p* = 0.059	*r* = − 0.510 *p* = 0.002	*r* = − 0.482 *p* = 0.005	*r* = − 0.576 *p* = 0.001	*r* = 0.803 *p* = 0.001	*r* = 0.790 *p* = 0.001

*DFT*, Dubousset Functional Test; *FRT*, Functional Reach Test; *BBS*, Berg Balance Scale; *3MBWT*, 3-m backward walk test; *UPDRS-III*, Unified Parkinson’s Disease Rating Scale-motor function; *POMA-G*, Tinetti Performance-Oriented Mobility Assessment-Gait; *POMA-B*, Tinetti Performance-Oriented Mobility Assessment-Balance; *POMA-T*, Tinetti Performance-Oriented Mobility Assessment-Total; *TUG*, Timed Up and Go Test

## Discussion

To assess the functionality and applicability of an implementation in academic studies and clinical practices, test result measurements should be valid, reliable, and sensitive to changes in neurologic patients. The current study contributed significantly to the literature in terms of determining the validity and reliability of the DFT in early stage PD patients. The DFT was found to be valid and reliable in early stage PD patients. Moreover, this study aimed to calculate the SEM and MDC values of the DFT. Accordingly, it is aimed to help clinicians observe the clinical course of early stage PD patients with more objective values in practical use. These values will contribute significantly to experts, who play an active role in the rehabilitation process in terms of follow-up of early stage PD patients in clinical practice, since they change at minimally significant levels.

The Timed Up and Go (TUG) test is a simple, fast, and extensively used clinical tool for assessing lower extremity function, mobility, and fall risk. The TUG test involves many activities that are common in daily life, such as sitting-standing up, walking, and turning [20]. It has been defined as a valid and reliable measure of mobility in PwPD [38], and both the American Geriatrics Society and the British Geriatrics Society recommend this test as a component of multifactorial fall risk assessment [39]. The validity and reliability study of the test in PwPD was conducted by Morris et al. [38], and the reliability of the test–retest is excellent in PwPD (ICC = 0.99). Huang et al. [28] have demonstrated excellent test–retest (ICC ¼ 0.80) reliability in PwPD. However, this test primarily assesses balance and mobility, particularly walking forward and the ability to turn back. The up and walking test, which is the component of the DFT, is similar to the TUG test, but it also evaluates the balance and mobility of individuals while walking backward. Backward walking is more difficult and necessitates more reliance on neuromuscular control, proprioception, and protective reflexes [40]. Recent studies have revealed that the evaluation of backward walking provides better diagnostic accuracy in evaluating mobility and balance disorders [40]. The validity and reliability study of the 3MBWT was performed by Koçer et al. [31], and the reliability value was determined to be excellent (ICC = 0.965). The up and walking test (ICC = 0.819) for older adults [41], (ICC = 0.939) for all stroke patients, (ICC = 0.938) for patients have 6–12-month stroke duration, and (ICC = 0.932) for patients have 12 months and more stroke duration was found to have excellent intrarater reliability [42]. Similarly, in this study, the up and walking test (ICC = 0.952) was found to have excellent intrarater reliability in early stage PD patients. In our study, the TUG, dual-task TUG, POMA, FRT, BBS, and 3MBWT, which are frequently used in the clinic, were used to test the validity of the DFT up and walking test. According to the correlation analysis, a high level of positive correlation was found between the TUG, dual-task TUG, and 3MBWT, a high level of negative correlation was found between the BBS, and a moderate negative correlation was revealed between the POMA-B, POMA-G, POMA-T, and FRT scores. Since only dynamic forward balance is evaluated in the FRT test and forward walking and balance parameters are evaluated in the POMA test, we think that this situation is reflected in our results. The time taken to complete the up and walking test had moderate correlation with UPDRS-III. In line with our findings, mobility, balance, and gait speed, which are associated with the up and walking test according to our findings, were correlated with these variables. Bradykinesia, rigidity, and balance components of the UPDRS-III were related to up and walking test performance. According to these results, we consider that the DFT up and walking test is a more functional, useful, and highly advantageous test to be applied in early stage PD patients since it evaluates both the forward walking function and functionality during backward walking, thus providing two test features for the use of clinicians.

Stair climbing is one of the most difficult motor activities of daily living. The ability to ascend the steps safely is a significant component of the skills needed to maintain mobility and independence, both at home and in society. A new study based on a major National Health Interview Survey defined stairs and steps as one of the three most common hazards associated with fall injuries within all age groups [43, 44]. Parkinson’s patients (PD) often report difficulties in climbing stairs or falling downstairs. Ascending stairs requires precise coordination of alternative lower extremity movements for correct placement of the foot on each step, a high level of stability control for single lower extremity balance during lower extremity advancement, and voluntary control to obtain visual information about the characteristics of the stairs. In the literature, the number of tests evaluating postural stability while ascending stairs is very low, and no validity and reliability studies of these tests in Parkinson’s patients have been encountered [45].

The DFT step test was found to have an excellent agreement between test–retest reliability: ICC = 0.935 for older adults [41], ICC = 0.973 for all stroke patients, ICC = 0.976 for patients who have 6–12-month stroke duration, and ICC = 0.966 for patients who have 12-month and more stroke duration [42]. Excellent interrater and test–retest reliability (ICC = 0.955) were found in people with PD, which was in line with that reported in stroke and older adults. There was a high level of correlation between the DFT step test and TUG, dual-task TUG, and the 3MBWT and a moderate correlation between the POMA-B, POMA-G, POMA-T, BBS, UPDRS-III, and FRT. Because the TUG and 3MBWT are rather scales that have the characteristics of dynamically evaluating balance and mobility during walking, we are of the opinion that the values and correlation coefficients obtained in the step test in our study are reflected in the results of our study. The FRT, on the other hand, affected our results since it is a test that evaluates dynamic balance during forward bending. Since the BBS and POMA that evaluate static-dynamic balance, not only gait, which could explain the relationship between the step test and the BBS and POMA. The step test had moderate correlation with UPDRS-III. These findings may indicate that motor symptoms deteriorate the step skill of early stage PD patients, which adversely affects daily living activities.

Sitting and standing up from the ground is a functional task necessary for actions such as cleaning the house in activities of daily living, and if a fall occurs, it is crucial to return to the upright position. The transitions from sitting to standing and from standing to sitting are components of some daily functional tasks that are quite difficult in terms of postural control [46]. Transition from a standing position to a sitting position on the floor or vice versa requires an appropriate level of muscle strength, coordination, balance, and flexibility, and these are usually impaired in Parkinson’s patients [47]. Anticipatory postural adjustments (APAs) are included in the sitting and standing performance. Planning of APAs involves various structures of the central nervous system (CNS), such as the pre-motor cortex, complementary motor area, basal ganglia, and cerebellum. These structures transmit information to the pedunculopontine nuclei, which are important to modulate APAs, via independent channels [48, 49]. The neural connection between the basal ganglion and the pedunculopontine nucleus is impaired in PwPD, resulting in postural control deficiencies. Therefore, postural control and stability problems arise in activities of sitting and standing up from the ground in PwPD [48]. It is quite important to assess this activity, which has an important place in activities of daily living. In the literature, the most commonly used tests of sitting and standing movement are the five-time sit-to-stand test and the 30-s sit-to-stand test [50]. However, these tests evaluate the performance of sitting and standing up from the chair, while the DFT down and sitting test evaluates the performance of transition from the standing position to the sitting position on the ground and transition back to the standing position.

Excellent interrater and test–retest reliability were found in early stage PD patients (ICC = 0.917), which was in line with that reported in stroke [42] and older adults [41]. A moderate correlation was found between the DFT down and sitting test and TUG, 3MBWT, POMA-B, POMA-G, POMA-T, FRT, BBS, UPDRS-III, and dual-task TUG. Impaired down and sitting ability may be affected by increased motor symptoms, severity, and stage of the disease. Consequently, these impairments may result in difficulties in activities of daily living. The results demonstrate that the DFT down and sitting test is preferable to other tests since it evaluates sitting-standing ability, which is a part of activities of daily living.

Many activities of daily living require individuals to perform more than one task simultaneously, which is called a dual task. There is a need for adequate balance, coordination, attention, and judgment when performing dual tasks that require motor and cognitive systems to work together. Individuals with PD have been demonstrated to be significantly more sensitive to balance changes when performing dual tasks compared to controls of the same age [51]. These changes include a decrease in automaticity and impaired attention flexibility due to cortical and basal ganglia dysfunction. They cause balance disorders and increase the risk of falls. In the literature, it has been revealed that the TUG test, which includes cognitive tasks (TUG-cognitive) (three rhythmic backward counting), is more sensitive and specific in predicting the rate of falls in PwPD [29]. Thus, an emphasis is laid on the importance of evaluating the interaction between cognition and mobility while performing dual-task activities during the balance task in PwPD.

The DFT dual-tasking test showed an excellent test–retest reliability (ICC = 0.919) for early stage PD patients. Similar results were reported in reliability studies performed in other populations such as stroke [42], community-dwelling older adults [41]. The DFT dual-tasking test was highly correlated with the TUG, dual-task TUG, and 3MBWT while moderately correlated with the FRT, BBS, UPDRS-III, POMA-B, POMA-G, and POMA-T. The DFT dual-tasking test seems to be preferable to other tests since it evaluates the dual-task ability, which is a part of activities of daily living. None of the other tests used in the literature that measure gait and balance includes dual tasks. This test also requires cognitive dual task and cognitive capacity. Because it involves complex motor-cognitive abilities and evaluates many parameters, it can be said that the use of the DFT test is more advantageous in Parkinson’s patients compared to other tests. It is clear that the correlation between balance and dual-task performance must be considered in Parkinson’s patients to determine balance, functional independence, and risk of falls, prevent falls, and plan rehabilitation programs.

MDC and SEM values are extremely important for clinicians in clinical practice. While SEM was used to determine the possible error associated with Parkinson’s patients’ scores, MDC was used to interpret the clinical significance of the score obtained. Physiotherapists should expect DFT test parameters to be above 1.45, 1.39, 1.70, and 1.57, respectively, to see that physiotherapy and rehabilitation programs are effective in early stage PD patients. With a change above these values, we can say that minimal significant clinical gains are possible during the rehabilitation programs of early stage PD patients.

The present study has some limitations. First, the majority of all Parkinson’s patients were in mild to moderate stages of the disease, and there were only seven PwPD in H&Y stage 1.5 and seven people in H&Y stage 2, and four people in H&Y stage 2.5, and 15 people in H&Y stage 3. In our sample, there were no patients in H&Y stage 4 (advanced PD). Advanced Parkinson’s disease is characterized by progressive dopaminergic denervation leading to dopaminergic motor and non-motor fluctuations. Fluctuations contribute to impairments in balance, gait, physical performance, and dual-task performance more than mild or moderate stages of disease. As a result, DFT test results will also be affected in advanced PD. This may limit the generalization of the results for all stage of PD. There is a need for more research to confirm the timed DFT test in individuals with disabilities hospitalized in the late stages of the disease and divided into subgroups according to the H&Y stage. Second, this study mainly focused on the time spent on the basis of DFT tests. Thus, future studies can be designed to examine other parameters that may influence test performance, such as muscle strength and proprioception. Third, Parkinson’s patients were in the “on” state. Studies can be conducted on the reliability and validity of the DFT test when patients are in the “off” state.

According to the results acquired from our study, we believe that the DFT can be used as an objective evaluation tool in the clinic to evaluate the characteristics of early stage PD patients, such as balance and functional mobility. It is important to eliminate the activity limitations via the early identification of postural instability in early stage PD patients, prescription of an appropriate exercise program, and walking aids. To this end, there is a need for clinically valid and reliable evaluation tests that can identify mild postural instability in early stage PD patients and are suitable for evaluating and comparing interventions from different specific balance rehabilitation programs in this population. We think that the DFT, whose validity and reliability in early stage PD patients have been revealed, is objectively guiding in evaluating dynamic and static balance along with functional performance in rehabilitation programs.
